# Trypophobia is predicted by disgust sensitivity, empathic traits, and visual discomfort

**DOI:** 10.1186/s40064-016-3149-6

**Published:** 2016-08-30

**Authors:** Shu Imaizumi, Manami Furuno, Haruo Hibino, Shinichi Koyama

**Affiliations:** 1 Graduate School of Arts and Sciences, University of Tokyo, 3-8-1 Komaba, Meguro, Tokyo, 153-8902 Japan; 2 Graduate School of Engineering, Chiba University, 1-33 Yayoi, Inage, Chiba, 263-8522 Japan; 3 Japan Society for the Promotion of Science, 5-3-1 Kojimachi, Chiyoda, Tokyo, 102-0083 Japan

**Keywords:** Emotion, Trypophobia, Disgust, Empathy, Vision, Individual differences, Questionnaire

## Abstract

Trypophobia refers to disgust for a cluster of objects, and is considered an extension of disgust for dangerous objects. Furthermore, trypophobic images possess certain spatial properties that can induce perceptually unpleasant states (i.e., visual discomfort). We examined whether trypophobia is associated with disgust sensitivity, empathic traits, and visual discomfort. Japanese adults (*n* = 126) completed four scales: the Trypophobia Questionnaire, which measures trypophobia proneness; the Disgust Scale-Revised, which measures disgust sensitivity; the Interpersonal Reactivity Index, which measures empathic traits; and the Visual Discomfort Scale, which measures proneness to visual discomfort. Trypophobia proneness was found to be predicted by Core disgust sensitivity (i.e., threat of disease), Personal Distress (i.e., the empathic trait of self-oriented emotional distress), and proneness to visual discomfort. We suggest a number of potential factors relating to individual differences in trypophobia and the possible cognitive and perceptual mechanisms underlying trypophobia.

**Electronic supplementary material:**

The online version of this article (doi:10.1186/s40064-016-3149-6) contains supplementary material, which is available to authorized users.

## Background

Trypophobia is the disgust response or unpleasant feelings—and often somatic responses (e.g., goosebumps)—induced by observing a cluster of innocuous objects (e.g., lotus seed pods) (Cole and Wilkins 2013). Trypophobia has yet to be listed in official classifications such as the Diagnostic and Statistical Manual of Mental Disorders (American Psychiatric Association 2013). Notably, the first study on trypophobia (Cole and Wilkins 2013) indicated a variability in individuals’ proneness to it, with 46 of 286 adults reporting aversion or unpleasantness in response to a trypophobic image while the others showed no response. A subsequently developed psychometric scale of trypophobia proneness confirmed these individual differences (Le et al. 2015). However, little is known about the psychological factors underlying trypophobia or its variability; thus, we explored the predictors of trypophobia in an attempt to identify these factors.

From an evolutionary perspective, trypophobia may be an extension of intrinsic disgust for scars, sores, and poisonous animals with spots, which may help in avoiding disease and germs (Cole and Wilkins 2013; Skaggs 2014). Disgust plays an important role in helping us avoid offensive situations (Rozin et al. 2008) and varies substantially among nonclinical individuals (Haidt et al. 1994; Rozin et al. 1999). This individual variability in disgust proneness is termed disgust sensitivity, and can be psychometrically assessed (Haidt et al. 1994; Olatunji et al. 2007b). Disgust sensitivity comprises three domains (Olatunji et al. 2007b): disgust for offensiveness and threat of disease (i.e., Core disgust), for stimuli serving as reminders of humans’ animal origins (i.e., Animal Reminder disgust), and for threat of transmission of contagion (i.e., Contamination disgust). As such trypophobia proneness, if related to avoidance of disease, might also relate to disgust sensitivity, particularly Core disgust.

Given the above cognitive account (Cole and Wilkins 2013; Skaggs 2014), if trypophobia is an extension of disgust for reminder of disease or danger, a disposition to project oneself into potentially threatening and dangerous situations or to perceive such situations and stimuli orienting towards oneself might be associated with trypophobia proneness. Empathic traits, which comprise four factors (Davis 1980, 1983), are the abilities to project oneself into others’ situations (i.e., Perspective Taking), transpose oneself imaginatively into the feelings of fictional characters (i.e., Fantasy), and share others’ emotions, whether other- or self-oriented (i.e., Empathic Concern or Personal Distress, respectively). We propose that trypophobia proneness may relate to empathic traits, especially Perspective Taking, Empathic Concern, and Personal Distress.

A *perceptual* account for trypophobia has also been proposed. Trypophobic images likely possess enhanced mid-spatial frequency properties (Cole and Wilkins 2013; Le et al. 2015), which can similarly be found in natural or artificial images capable of inducing visual discomfort (Fernandez and Wilkins 2008; O’Hare and Hibbard 2011), such as somatic unpleasantness, eyestrain, and perceptual distortions (Wilkins 1995). Since healthy individuals exhibit individual differences in visual discomfort (Conlon et al. 1999), there might be a linkage between the propensities for visual discomfort and trypophobia.

With the above background, we hypothesized that trypophobia proneness is associated with disgust sensitivity, emotional empathic traits, and visual discomfort.

## Methods

### Participants

One hundred twenty-six adults whose first language was Japanese (83 males, 43 females, mean age 39.72 years, *SD* = 9.41) participated in this study on April 2015. Age did not differ between the genders [*t*(124) = .14, *p* = .89, *d* = .03]. Participants were recruited using Yahoo! Crowdsourcing, an online labor market similar to Amazon Mechanical Turk, whose validity and reliability for psychometric studies have been confirmed (Buhrmester et al. 2011; Shapiro et al. 2013).

### Measures

The Trypophobia Questionnaire (TQ; Le et al. 2015), Japanese version (Imaizumi et al. 2016) comprises 17 items with a one-factor structure assessing proneness to emotional (e.g., “Feel aversion, disgust or repulsion”) and somatic responses (e.g., “Have goosebumps”) induced by trypophobic stimuli (e.g., lotus seed pods, honeycombs). Participants rated their agreement with each item on a five-point scale ranging from 1 (“not at all”) to 5 (“extremely”). Responses were summed to produce a score of trypophobia proneness.

The Disgust Scale-Revised (DS-R; Haidt et al. 1994; modified by Olatunji et al. 2007b), Japanese version (Iwasa and Tanaka 2013), comprises 25 items measuring disgust sensitivity in three subscales: Core, Animal Reminder, and Contamination disgust. Participants indicated their agreement with 13 statements on a five-point scale ranging from 0 (“strongly disagree”) to 4 (“strongly agree”), and evaluated how disgusting they would find 12 situations, also on a five-point scale (0 = “not at all disgusting” to 4 = “extremely disgusting”). Although the original version adopted two- and three-point scales, five-point scales were used based on advice from the original authors of the English scale. Subscale scores were calculated by summing item scores.

Emotional and cognitive empathic traits were assessed with the Interpersonal Reactivity Index (IRI; Davis 1980, 1983), Japanese version (Sakurai 1988), which comprises 28 items in four subscales: Perspective Taking, which assessed cognitive empathic traits; and Fantasy, Empathic Concern, and Personal Distress, which assessed emotional empathic traits. Each item was answered using a five-point scale ranging from 0 (“does not describe me well”) to 4 (“describes me very well”). The empathic traits were defined as the summed item scores of each subscale.

We used the 23-item Visual Discomfort Scale (VDS; Conlon et al. 1999) to assess daily experiences of visual discomfort, including abnormal perception and somatic symptoms. Each item was answered using a four-point scale ranging from 0 (“event never occurs”) to 3 (“almost always”). The sum of item scores indicated proneness to visual discomfort.

### Procedure

This survey was administered via the online tool SurveyMonkey (http://www.surveymonkey.com) using participants’ own computers. Initially, potential participants were informed that participation was voluntary and that they could quit any time. Individuals who consented to participate first completed the TQ, then the DS-R, IRI, and VDS in a random order, and reported their gender and age. Finally, participants were thanked and paid 150 Japanese yen (approximately 1.25 US dollars).

### Data analysis

We analyzed descriptive statistics and zero-order Pearson correlations for the TQ, DS-R subscales, IRI subscales, and VDS. Next, we checked for gender differences in these scales using two-tailed *t* tests because females may score higher on disgust sensitivity (Olatunji et al. 2008) and empathic traits (Davis 1980; Mestre et al. 2013) than males. To identify variables predicting trypophobia proneness, we performed stepwise multiple regression analysis with TQ as the dependent variable, and the DS-R subscales, IRI subscales, VDS, and gender as independent variables. All analyses were conducted using SPSS Statistics 20.0 (IBM Corp., Armonk, NY, USA) with the significance level set at *p* < .05.

## Results

Table 1 displays descriptive statistics, correlations, and Cronbach’s alphas of the current sample. TQ positively correlated with Core disgust sensitivity, Personal Distress, and VDS. Besides the within-scale positive correlations for the DS-R and IRI, we found that Core disgust negatively correlated with Perspective Taking and Empathic Concern. Core disgust sensitivity was higher in women [*M*_men_ = 18.47, *SD*_men_ = 5.72, *M*_women_ = 21.44, *SD*_women_ = 5.44, *t*(124) = 2.81, *p* < .01, *d* = .53]; no other gender differences were found (|*t*|s ≤ 1.44, *p*s ≥ .15, *d*s ≤ .26).

**Table 1:** Descriptive statistics and Pearson’s correlations

	Mean	*SD*	1	2	3	4	5	6	7	8	9
1. TQ	28.35	11.18	(.92)								
2. Core	19.48	5.78	.30***	(.83)							
3. Animal reminder	10.37	2.11	.15	.45***	(.71)						
4. Contamination	11.15	4.27	.17	.55***	.40***	(.69)					
5. Perspective taking	22.69	4.22	−.06	−.24**	−.14	−.10	(.79)				
6. Fantasy	22.37	4.89	.05	−.04	−.03	−.01	.38***	(.77)			
7. Empathic Concern	22.78	3.11	−.12	−.19*	−.05	−.08	.42***	.40***	(.52)		
8. Personal distress	20.79	4.73	.29**	.14	−.01	.06	−.15	.06	−.07	(.79)	
9. VDS	9.31	8.71	.28**	.09	−.14	.07	.02	.17	−.03	.13	(.94)

*N* = 126. Cronbach’s alphas in parentheses

*TQ* Trypophobia Questionnaire, *VDS* Visual Discomfort Scale

* *p* < .05; ** *p* < .01; *** *p* < .001

Table 2 displays the regression results predicting TQ scores. The residuals in the analysis were independent (Durbin-Watson index, 1.98), and the predictors’ variance inflation factors for all steps were <1.42, indicating no issue of multicollinearity. In the final step, Core disgust, Personal Distress, and VDS all positively predicted TQ.

**Table 2:** Stepwise multiple regression predicting Trypophobia Questionnaire score

Predictors	*R* ^2^	Adjusted *R* ^2^	*ΔR* ^2^	*F*	*B* (SE)	*β*	*t*	VIF
Step 1	.09	.08	.09	11.83**				
Core disgust					.57 (.17)	.30	3.44**	1.00
Step 2	.15	.14	.07	11.00***				
Core disgust					.53 (.16)	.27	3.25**	1.01
VDS					.33 (.11)	.26	3.06**	1.01
Step 3	.20	.18	.05	10.20***				
Core disgust					.47 (.16)	.24	2.96**	1.03
VDS					.29 (.11)	.23	2.79**	1.02
Personal distress					.53 (.20)	.23	2.73**	1.04

*N* = 126

*VDS* Visual Discomfort Scale, *SE* standard error, *VIF* variance inflation factor

** *p* < .01; *** *p* < .001

## Discussion

We explored the predictors of trypophobia proneness, as measured by the TQ (Le et al. 2015), among Japanese adults. The significant predictors included Core disgust sensitivity, Personal Distress, and proneness to visual discomfort. Moreover, we observed no gender differences in trypophobia proneness, consistent with our previous study (Imaizumi et al. 2016); Core disgust, however, was higher among females, which accords with previous studies (Olatunji et al. 2008).

The predictive effect of Core disgust sensitivity is logical, given that it represents proneness to perceiving a threat of disease for stimuli such as waste products and small animals (Olatunji et al. 2007b; Rozin et al. 2008). In other words, it may partially overlap with disgust and discomfort elicited by trypophobic stimuli which displays a clusters of small objects and perhaps an appearance of poisonous animals (Cole and Wilkins 2013). Similarly, trypophobia propensity was also predicted by Personal Distress—namely, the proneness to have self-oriented feelings of anxiety and unease (Davis 1980, 1983) and to desire to reduce one’s own uneasiness and disgust (Batson et al. 1983). If trypophobia is an extension of disgust for dangerous animals and skin lesions (Cole and Wilkins 2013; Skaggs 2014), then trypophobic stimuli, being threats of danger and disease, might serve as self-oriented negative stimuli for individuals with high proneness to trypophobia. Thus, we hypothesize that, ecologically, increased emotional functioning that facilitates avoidant behavior with respect to potential threats (Rozin et al. 2008) results in trypophobia. Given evidence that behaviorally measured disgust sensitivity (e.g., how close participants are willing to approach disgust elicitors; Rozin et al. 1999; Olatunji et al. 2007a) and personal distress (e.g., prosocial behavior and heart rate variation while observing needy others; Eisenberg et al. 1989) may correlate with their questionnaire measures, further research combining behavioral and psychometric measures may further enhance our understanding of the relationships between trypophobia, disgust, and empathy.

We found an association between trypophobia proneness and visual discomfort, both of which are induced by stimuli showing excessive energy at medium spatial frequencies that are unnatural and physiologically stressful for human vision (Fernandez and Wilkins 2008; Cole and Wilkins 2013; Le et al. 2015). A previous event-related potential study suggested that a larger amplitude of early posterior negativity, which reflects differential processing of emotional compared to neutral stimuli (Schupp et al. 2006), was evoked by trypophobic images selectively in the occipital area, implying that trypophobia results from low-level visual properties (Van Strien and Van der Peijl 2015). Thus, trypophobia may be triggered by both the cognitive (i.e., disgust) and physical (i.e., visual discomfort) properties of visual stimuli. Neuropsychological evidence locating disgust in the insula and basal ganglia (Calder et al. 2001; Murphy et al. 2003) and visual discomfort in the early visual cortices (Huang et al. 2003, 2011) can support this notion. However, it remains unclear whether and how disgust and visual discomfort derived from trypophobia interact. As there is evidence that individuals with migraine headaches are prone to visual discomfort (Marcus and Soso 1989) due to their cortical hyperexcitability (Huang et al. 2003), they might be also prone to trypophobia. Comparison of trypophobia proneness between individuals with and without migraine may help clarify the association of the visual discomfort and disgust components of trypophobia.

The determination coefficients in our multiple regression were relatively low, suggesting that other psychological factors may contribute to trypophobia. Neuroticism, one of the components of the Big Five personality traits, has been shown to predict disgust sensitivity, suggesting that disgust-sensitive individuals might be sensitive to general emotional stimuli and psychological distress (Druschel and Sherman 1999). This may be consistent with our results indicating relationships among trypophobia, Core disgust sensitivity, and Personal Distress. Moreover, studies have suggested that proneness to trypophobia weakly correlates with general anxiety (Le et al. 2015) and is predicted by social anxiety (Chaya et al. 2016). Therefore, further exploration of potential psychological and psychiatric factors of trypophobia is needed.

Findings of the current study were obtained from the online labor market in Japan. Replication using traditional paper-and-pencil surveys would strengthen the findings. Moreover, given that the factor structure of disgust sensitivity in a Japanese sample might differ from that in European, American, and Australian samples (Olatunji et al. 2009), investigation of trypophobia in various cultural groups will help to identify its cultural and ecological origins.

## Conclusions

We replicated previously found individual differences in trypophobia (Cole and Wilkins 2013; Le et al. 2015), and provided preliminary evidence for the predictors of trypophobia proneness. Future studies might attempt to elucidate the mechanisms underlying trypophobia to aid in developing interventions and to determine whether trypophobia is a full-blown psychiatric condition.

## Additional file

10.1186/s40064-016-3149-6 The raw questionnaire data.

## Abbreviations

TQ: Trypophobia Questionnaire
DS-R: Disgust Scale-Revised
IRI: Interpersonal Reactivity Index
VDS: Visual Discomfort Scale
